# Bacterial community composition in the salivary glands of triatomines (Hemiptera: Reduviidae)

**DOI:** 10.1371/journal.pntd.0006739

**Published:** 2018-09-13

**Authors:** Michele Souza Lima, Marinella Silva Laport, Elias Seixas Lorosa, José Jurberg, Kátia Regina Netto dos Santos, Mário Alberto Cardoso da Silva Neto, Caio Tavora Coelho da Costa Rachid, Georgia Correa Atella

**Affiliations:** 1 Instituto de Bioquímica Médica Leopoldo de Meis, Universidade Federal do Rio de Janeiro, Rio de Janeiro-RJ, Brazil; 2 Instituto Nacional de Ciência e Tecnologia em Entomologia Molecular–INCT–EM, Universidade Federal do Rio de Janeiro, Rio de Janeiro-RJ, Brazil; 3 Instituto de Microbiologia Prof. Rogério Góes, Universidade Federal do Rio de Janeiro, Rio de Janeiro-RJ, Brazil; 4 Laboratório de Referência Nacional e Internacional de Triatomíneos, Instituto Oswaldo Cruz-Fiocruz, Rio de Janeiro-RJ, Brazil; Centro de Pesquisas René Rachou, BRAZIL

## Abstract

**Background:**

Chagas disease is caused by the parasite *Trypanosoma cruzi* and is transmitted through triatomines (Hemiptera: Reduviidae). In the last year, many studies of triatomine gut microbiota have outlined its potential role in modulating vector competence. However, little is known about the microbiota present in the salivary glands of triatomines. Bacterial composition of salivary glands in selected triatomine species was investigated, as well as environmental influences on the acquisition of bacterial communities.

**Methodology/Principal findings:**

The diversity of the bacterial communities of 30 pairs of salivary glands of triatomines was studied by sequencing of the V1- V3 variable region of the 16S rRNA using the MiSeq platform (Illumina), and bacteria isolated from skin of three vertebrate hosts were identified based on 16S rRNA gene sequence analysis (targeting the V3–V5 region). In a comparative analysis of microbiota in the salivary glands of triatomine species, operational taxonomic units belonging to *Arsenophonous* appeared as dominant in *Triatoma* spp (74% of the total 16S coverage), while these units belonging to unclassified Enterobacteriaceae were dominant in the *Rhodnius* spp (57% of the total 16S coverage). Some intraspecific changes in the composition of the triatomine microbiota were observed, suggesting that some bacteria may have been acquired from the environment.

**Conclusions and significance:**

Our study revealed the presence of a low-diversity microbiota associated to the salivary glands of the evaluated triatomines. The predominant bacteria genera are associated with triatomine genera and the bacteria can be acquired in the environment in which the insects reside. Further studies are necessary to determine the influence of bacterial communities on vector competence.

## Author summary

Chagas disease is caused by the parasite *Trypanosoma cruzi* and is transmitted through triatomines (Hemiptera: Reduviidae). It is estimated that over 10 000 people die every year from clinical manifestations of Chagas disease, and more than 25 million people risk acquiring the disease per year. Vector control remains the most effective method to prevent infection. In previous studies, the microbiota affected vector competence, thereby highlighting its potential for vector control. In this study, we demonstrate the presence of cultivable and non-cultivable bacteria in the salivary glands of different species of triatomines. The predominant bacterial genera appear to be specific to certain triatomines, e.g., the operational taxonomic units belonging to *Arsenophonus* bacterial genus is associated with the *Triatoma* spp, while these units belonging to unclassified Enterobacteriaceae bacterial family are associated with the *Rhodnius* spp. The operational taxonomic units found in low relative abundance also varied between species of triatomines and their occurrence could be influenced by the environment in which insects reside as well as inter-bacterial modulation by species-specific manner.

## Introduction

In the last few years, multiple studies have focused on understanding the role of microbiota in vector competence, due to the ability of microbiota to acquire, maintain and modulate of pathogens transmission [1, 2]. Bacterial communities can establish different interactions with insects, such as commensal, mutual or pathogenic relationships. Establishment of the microbiota depends on the environmental conditions in which the insect is found, as well as the tolerance of the microbiota to the insect´s immune system [3]. In the gut, bacteria have an important role in supplying essential nutrients to the insect, facilitating digestion and defending the gut against opportunistic pathogens, such as parasites and other bacteria [4, 5].

Several studies have demonstrated the influence of environmental conditions on bacterial communities. Variability in insect-associated microbiota has been detected among mosquitoes from different geographic locations and between field and lab populations [6, 7, 8]. *A*. *stephensi* mosquitoes raised in the lab showed reduced bacterial diversity in their midguts compared to field-caught mosquitoes. Similarly, *A*. *gambiae* mosquitoes raised in the lab possessed 45 distinct Operational Taxonomic Units (OTUs) of bacteria, compared to 155 OTUs in field-caught mosquitoes. Differences among bacterial taxa richness in field-caught mosquitoes demonstrate the extent to which bacteria are acquired from the habitat [9, 10].

More than 57 species of cultivable bacteria have been identified in the triatomine gut [11]. Some culture-independent methods have also revealed the microbiota composition in the gut of triatomines and interactions with *T*. *cruzi* and *T*. *rangeli* parasites [12, 2]. The bacterial communities present in the salivary glands of some species of ticks (*Ixodes ovatus*, *I*. *persulcatus* and *Haemaphysalis flava*) and mosquitoes (*Anopheles gambiae*, *A*. *culicifacies* and *A*. *stephensi*) have been characterized and shown to be species-specific [13, 14, 15]. However, the bacterial communities in triatomine salivary glands have never been investigated.

In this study was characterized for the first time, the presence of different cultivable and non-cultivable bacteria in the salivary glands of triatomines. Environmental influences on microbiota composition were also evaluated through the collection of insects in different localities. Furthermore, the antimicrobial production of cultivable bacteria present in the salivary glands of *T*. *infestans* was tested in order to establish a relationship between insects and bacteria colonization.

## Materials and methods

### Ethics statement

The animal care and experimental protocols were conducted following the guidelines of the institutional care and use committee (Committee for Evaluation of Animal Use for Research from Federal University of Rio de Janeiro, CAUP-UFRJ) and the NHI Guide for the Care and Use of Laboratory Animals (ISBN 0_309_05377_3). The protocols were approved by CAUP-UFRJ under registry #BQM001 and #IBQM027. Technicians dedicated to the animal facility carried out all aspects related to animal husbandry under strict guidelines to ensure their careful and consistent handling. All human participants involved in this study gave their written consent.

### Insects

Eighty-five insects in their adult stages, including males and females, were obtained from different endemic areas in South America and insectary specimens. The insects were maintained at 28°C with 60–80% relative humidity and fed at 3-week intervals. The geographic origin of each triatomine and insectary are given in Table 1 and S1 Fig.

**Table 1:** Triatomine species, institute and period of their maintenance, host for feeding and number of specimes.

Species	Institute of maintenance	Field caught	Period of maintenance in the insectary	Host for feeding	Number of specimens
***Rhodnius prolixus***	Fiocruz	-	< 5 years	Mice	5
***R*. *prolixus***	UFRJ	-	> 30 years	Rabbit	5
***R*. *brethesi***	Fiocruz	-	< 5 years	Mice	5
***R*. *milesi***	Fiocruz	-	< 5 years	Mice	5
***R*. *milesi***	UFRJ		< 2 years	Rabbit	5
***R*. *neglectus***	Fiocruz	-	< 5 years	Mice	5
***R*. *equatoriensis***	Fiocruz	-	< 5 years	Mice	5
***R*. *nasutus***	Ø	+	Ø	Ø	5
***Panstrongylus megistus***	Fiocruz	-	< 5 years	Mice	5
***Triatoma brasiliensis***	Fiocruz	-	< 5 years	Mice	5
***T*.*brasiliensis***	UFRJ	-	< 2 years	Rabbit	5
***T*.*rubrovaria***	Fiocruz	-	< 5 years	Mice	5
***T*.*rubrovaria***	UFRJ	-	< 2 years	Rabbit	5
***T*.*infestans***	Renne Rachou	+	< 5 years	Chicken	10
***T*.*infestans***	Fiocruz	+	< 6 years	Mice	10

### Isolation of bacteria from mammals

To verify whether the microbiota present in the triatomines´ salivary glands could have been acquired via contact with vertebrates, the bacteria found on the isolated from skin of the animals that served as food for insects and on the skin of the humans that handled them were isolated. The participants in the present study were technicians who worked in the insectaries of the research institutions (UFRJ and Fiocruz). In the insectary of Fiocruz, all personel have to wash their hands every 30 minutes to avoid infections in the insectary. One technician of each institution was chosen randomly for this study. The technicians were fit and healthy without any cuts or wounds on their hands and were informed about all aspects of the experiment. The participant´s hands were swabbed, beginning from the flexor aspect of wrist, across the palm and up all five fingers. The swabs were rolled over BHI-agar plates, under sterile conditions, that were then incubated at 37°C for 24–72 h.

Two rabbits and two mice used to feed insects were anesthetized via an intramuscular injection of ketamine (100 mg/kg of body weight) in the leg and the mouse back skin and rabbit ear skin were swabbed and the swabs rolled over BHI-agar plates, which were then incubated at 37°C for 24–72 h. All experimental procedures were conducted following the guidelines of the institutional care and use committee described previously.

### Salivary gland dissection and incubation of bacteria

Salivary glands of *Rhodnius prolixus*, *R*. *nasutus*, *R*. *brethesi*, *R*. *milesi*, *R*. *neglectus*, *R*. *equatoriensis*, *Panstrongylus megistus* and *T*. *infestans* obtained from different places were dissected 7 days after insect feeding. Manipulations were come out on a sterile glass slide containing phosphate buffered saline (PBS, pH 7.4) by pulling off the insect head under a stereo microscope. Pairs of salivary glands were isolated in a drop of 1x PBS, cleaned of any adhering tissue, rinsed twice in sterile PBS, transferred to a microcentrifuge tube and macerated with the help of homogenizers for 30s. The samples were centrifuged at 2000 x g for 15 min and supernatant containing the salivary gland content of each insect was transferred onto BHI-agar plates, incubated at 28°C for 24–72 h. All steps were performed under aseptic conditions.

### Bacterial isolates and 16S rRNA sequence analysis

Colony-forming units (CFU) were selected based on size and colony appearance (smooth or rough) and presence of pigments, as an attempt to cover all colony morphologies observed. Phenotypic characterization of the bacterial isolates was performed using microbiological data, colony morphology and coloration plus Gram-staining. From each agar plate containing salivary gland material, four bacterial isolates with the same characteristics were purified in slant cultures and stored at– 80°C. Only one isolate of each morphotype was identified by 16S rRNA sequence analysis. Bacterial DNA was recovered by a thermal lysis protocol consisting in resuspending each colony in 25 μl sterile PCR water and boiling the suspension at 100°C for 15 min. PCR amplification was performed by adding 1.5 μl DNA solution to 23.5 μl of mix containing 1x buffer GOTAQ Green master mix (Promega), 0.4mg/ml of BSA (Sigma), 0.05% of Igepal (Sigma), and 20 pmol of each universal primer, 27F (5′-GAGTTTGATCMTGGCTCAG-3′) and 1492R (5’-GGYTACCTTGTTAACGACTT-3’) [16]. The PCR reaction was conducted using the following conditions: An initial denaturation step at 94°C for 6 min was followed by 30 cycles at 94°C for 30 s, 55°C for 1 min 30 s and 72°C for 2 min, and a final elongation step at 72°C for 5 min. PCR products were confirmed by electrophoresis on a 0.8% agarose gel, purified using the Agencourt AMPure XP (Beckman Coulter, USA) and sequenced using the universal primer 338F (5´-ACTCCTACGGGAGGCAGC-3´) by ABI3500 Genetic Analyzer (Applied Biosystems) at Biotecnologia, Pesquisa e Inovação LTDA- BPI company (Botucatu, SP). Each PCR product generated one sequence. All sequences were analyzed using the online portal of the SILVA SINA alignment service of the ARB-Silva database (http://www.arb-silva.de/aligner/) [17]. Sequence data have been deposited in the GenBank database under the respective accession numbers (Tables 1 and 2).

**Table 2:** Bacterial isolates from triatomine salivary glands, their origin, identity and GenBank ID.

Source of isolation	Bacterial species	Identity (%)	GenBank acession #
***Rhodnius nasutus***	*Gordonia polyisoprenivorans*	100	KX830836
***Rhodnius brethesi*** ^**c**^	*Rhodococcus rhodnii*	100	KX830837
***Rhodnius milesi*** ^**c**^	*Rhodococcus rhodnii*	100	KX830838
***Rhodnius neglectus*** ^**c**^	*Rhodococcus rhodnii*	100	KX830840
***Rhodnius prolixus*** ^**c**^	*Rhodococcus rhodnii*	100	KX830841
***Rhodnius equatorienses*** ^**c**^	*Rhodococcus rhodnii*	99.8	KX830842
***Panstrongylus megistus*** ^**c**^	*Enterococcus faecalis*	100	KX830839
***Triatoma infestans*** ^**b**^	*Enterococcus faecalis*	100	KX830843
***Triatoma infestans*** ^**b**^	*Proteus mirabilis*	99.8	KX830845
***Triatoma infestans*** ^**a**^	*Corynebacterium xerosis*	100	KX830848
***Triatoma infestans*** ^**a**^	*Rhodococcus rhodnii*	99.8	KX830849

a. Insects collected in the field and kept in Renne Rachou insectary

b. Insects collected in the field and kept in Instituto Oswaldo Cruz insectary

c. Insects from Instituto Oswaldo Cruz insectary

### Bacterial community analysis

#### DNA isolation

Salivary glands of *T*. *brasiliensis*, *T*. *infestans*, *T*. *rubrovaria*, *R*. *milesi* and *R*. *prolixus* obtained from Fiocruz and the UFRJ insectary were dissected and subjected to DNA isolation. For each composite sample, pairs of salivary glands from 30 insects were pooled. The biological replication was ensured by using different generations of insects for each composite sample. The DNA of salivary glands was isolated using the Ultra Clean Microbial DNA Isolation Kit (MO BIO Laboratories, USA) following the manufacturer´s protocol. DNA concentration was determined in a NanoDrop ND-1000 spectrophotometer (Thermo Fisher Scientific, USA) and the DNA samples were shipped to the MR DNA (www.mrdnalab.com, Shallowater, TX, USA) for the partial sequencing of the 16S rRNA gene.

### Primers and 16S rRNA gene amplification conditions

The V1- V3 variable region of the 16S rRNA was amplified by PCR using the primers 27Fmod (AGRGTTTGATCMTGGCTCAG) and 519Rmod (GTNTTACNGCGGCKGCTG) with barcode on the forward primer [18], using the HotStarTaq Plus Master Mix Kit (Qiagen, USA) under the following conditions: 94°C for 3 minutes, followed by 28 cycles of 94°C for 30 seconds, 53°C for 40 seconds and 72°C for 1 minute, after which a final elongation step at 72°C for 5 minutes was performed. After amplification, PCR products were separated in 2% agarose gel to determine the success of amplification and the relative intensity of bands. Multiple samples were pooled together in equal proportions based on their molecular weight and DNA concentrations. Pooled samples were purified using calibrated Ampure XP beads and then the pooled and purified PCR product was used to prepare a DNA library by following Illumina TruSeq DNA library preparation protocol. Paired-end Sequencing was performed at MR DNA (www.mrdnalab.com, Shallowater, TX, USA) on a MiSeq following the manufacturer’s guidelines.

### Bioinformatics analysis

The raw joined sequences were processed using Mothur v.1.39.1 software [19]. Sequences from both ends were joined with make.contigs command and the primers and barcodes list and with checkorient = t and pdiffs = 1. The sequences were then screened with scree.seqs command, removing the sequences with any ambiguity (maxambig = 0), large homopolymer (maxhomop = 8) short and very long reads (minlength = 450, maxlength = 550). The sequences were then aligned using a modified Silva database (across a virtual PCR with the same primers of the samples) as reference [20] and the resultant alignment were submitted to screen seqs and filter seqs to remove sequences with bad alignment and uninformative columns of the alignment. The sequences were then pre-clustered using the command pre.cluster with parameter diffs = 4. The chimeras were detected with the command chimera search and then eliminated. The sequences were classified using classify.seqs command, with RDP database [21] as reference and a bootstrap cutoff of 80. Sequences classified into chloroplasts, mitochondria, Eukarya, Archaea and those not assigned to any kingdom were removed. The resultant sequences was used as input for cluster.split command, using splitmethod = fasta and taxlevel = 4. It clustered all sequences into operational taxonomic units (OTUs), with a cutoff of 3% of dissimilarity. The sequences were further filtered to remove singletons. At this point, the number of sequences per sample varied from 6,785 to 68,236. To avoid bias due sampling effort, the samples were then randomly normalized to the same number of sequences (6,785). Then the taxonomy summary was used to understand the bacterial composition of each sample and the OTU distribution was used to calculate the diversity index, to establish the relationship between samples and to evaluate significant differences over specific OTUs among the triatomine species.

### Statistical analysis

Diversity and taxonomic composition graphics were constructed using Microsoft Excel software and formatted in PowerPoint. After testing for normality and homoscedasticity, statistical differences of the diversity and richness indexes were tested using ANOVA, followed by Tukey test in PAST 3.11 [22]. The relationship of the microbial structure of the different samples was assessed using non-metric multidimensional scaling, with Bray-Curtis distance. The effect of triatomine genera and local of incubation over microbial community were tested using a two way permanova in PAST 3.11. To identify the OTUs significantly altered in *Triatomine* genera, Indicator Species Analysis [23] was used, using Mothur v.1.39.1. An OTU was considered an indicator when both the indicator value was higher than 80 and the p-value lower than 0.05. A box-plot of the relative abundance of the selected OTUs was generated using PAST 3.11 and formatted using Adobe Illustrator.

### Nucleotide sequence accession numbers

The data generated were deposited in the NCBI Sequence Read Archive (SRA) and are available under accession number SRP119410.

### Antagonistic interactions among bacterial isolates

Antagonistic interactions of bacteria were tested in an antimicrobial substance production method previously described [24]. Therefore, the bacteria strains tested for antimicrobial substance production will be termed “producer” strains while those used as targets will be called “indicator” strains. One hundred thousand cells of each producer strain were spotted onto BHI-agar and incubated at 28°C until the colony diameter reached 8 mm. In parallel, each indicator strain was grown in liquid medium at 28°C for 24–72 h. Then, 10^5^ cells of each indicator strain were mixed with 3 ml of BHI soft agar and poured over the plates. The plates were incubated at 28°C for 24–72 h and the diameter of the inhibition zone around the spotted strain was measured. An indicator strain was considered sensitive to the activity of the producer strain when it exhibited a clear inhibition zone with a diameter > 8mm.

The bacteria isolates from *T*. *infestans* salivary gland were used for antagonistic interactions against indicator strains *Staphylococcus aureus* ATCC 29213 and *Escherichia coli* ATCC 25922. In addition, these isolates were screened against each other for antagonistic interaction to determine whether one would inhibit growth of the others.

## Results

### Isolation of cultivable bacteria from the salivary glands of different triatomine species

To verify whether the microbiota present in the triatomines´ salivary glands could be influenced by the environment or whether there would be a species-specific relationship between bacteria and insects, luminal contents of salivary glands from triatomines of different species and kept under different environmental conditions were inoculated on BHI agar. Five distinct bacterial strains were isolated and characterized based on colony morphology and coloration. In each salivary gland, only one CFU morphotype was observed and only *Proteus mirabilis* reacted Gram-negative, whereas the others reacted Gram-positive. Bacteria isolated from the salivary glands of *R*. *brethesi*, *R*. *milesi*, *R*. *neglectus*, *R*. *prolixus* and *R*. *equatorienses* were identified as *R*. *rhodnii* and the colonies developed a pink pigmentation (Table 2). Among the *Rhodnius* species collected in the field, *Gordonia polyisoprenivorans* was identified and developed an orange pigmentation. Different bacteria species were found in *T*. *infestans* collected in different localities (Table 2).

### Comparative analysis of microbiota in the salivary glands of triatomine species

**Fig 1:**
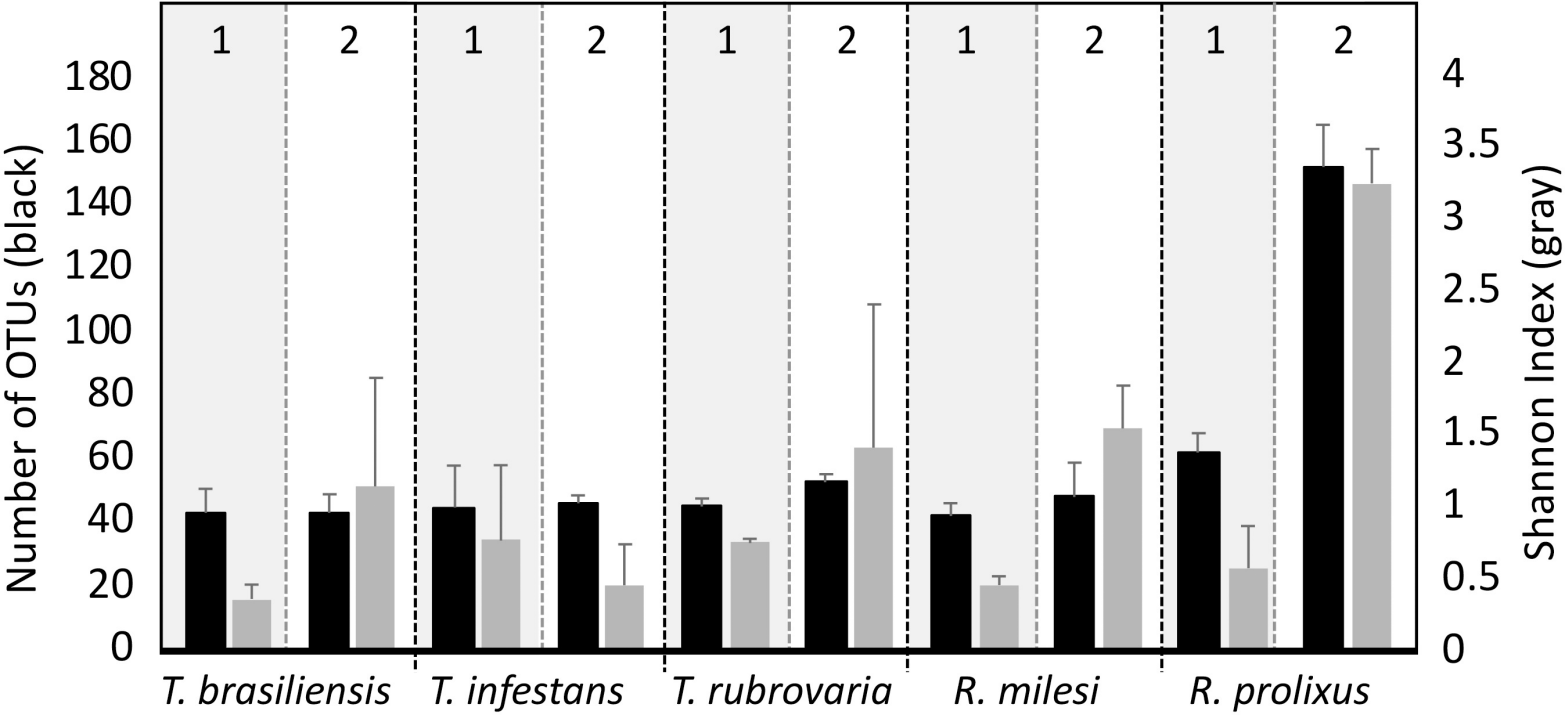
OTUs number (Black bars) and Shannon index (Gray bars) of the sequences obtained from *T*. *infestans*, *T*. *brasiliensis*, *T*. *rubrovaria*, *R*. *prolixus* and *R*. *milesi* salivary glands collected in Fiocruz (Insectary 1) and UFRJ (Insectary 2).

To characterize bacterial communities in the salivary glands of triatomine species, bacterial DNA was extracted and amplified with polymerase chain reaction of 16S rDNA. Thereafter, the amplicons were sequenced by next- generation sequencing technologies using the Illumina MiSeq platform. To assess the influence of the insectary on the microbial composition, the microbiome of the five different triatomine species were compared, each obtained from both UFRJ and Fiocruz insectaries. Bacterial richness found in the samples was low and relatively similar among species. The OTU count ranged from 40 to 60 per sample, with exception of the *R*. *prolixus* samples collected in the UFRJ insectary, which exhibited 150 OTUs. Statistical analysis of the bacterial OTUs revealed significant influences of both insectary (two-way ANOVA, *p* < 0.04) and species (two-way ANOVA, *p* < 0.01), with higher values found in UFRJ compared to Fiocruz and higher values found in *R*. *prolixus* compared to other triatomines (Fig 1). Bacterial diversity was quantified with the Shannon index. The analysis indicated a similar pattern to bacterial richness, with higher diversity found in the UFRJ insectary (*p* < 0.01) and a near-significant difference in diversity as a function of species (*p* = 0.056). Higher diversity was observed in *R*. *prolixus* (Fig 1). Detailed statistics of the sequencing database are summarized in S1 Table.

In the rarefaction curves of samples from *T*. *brasiliensis*, *T*. *infestans*, *T*. *rubrovaria*, *R*. *milesi* and *R*. *prolixus* (Fiocruz), the OTU coverage reached maximum saturation with fewer than 500 sequences. In samples from *R*. *prolixus* collected in the UFRJ insectary, the OTU coverage reached saturation with 3000 sequences. The rarefaction curve indicated sequencing depth was adequate to capture all bacterial communities present in the samples (S2 Fig).

**Fig 2:**
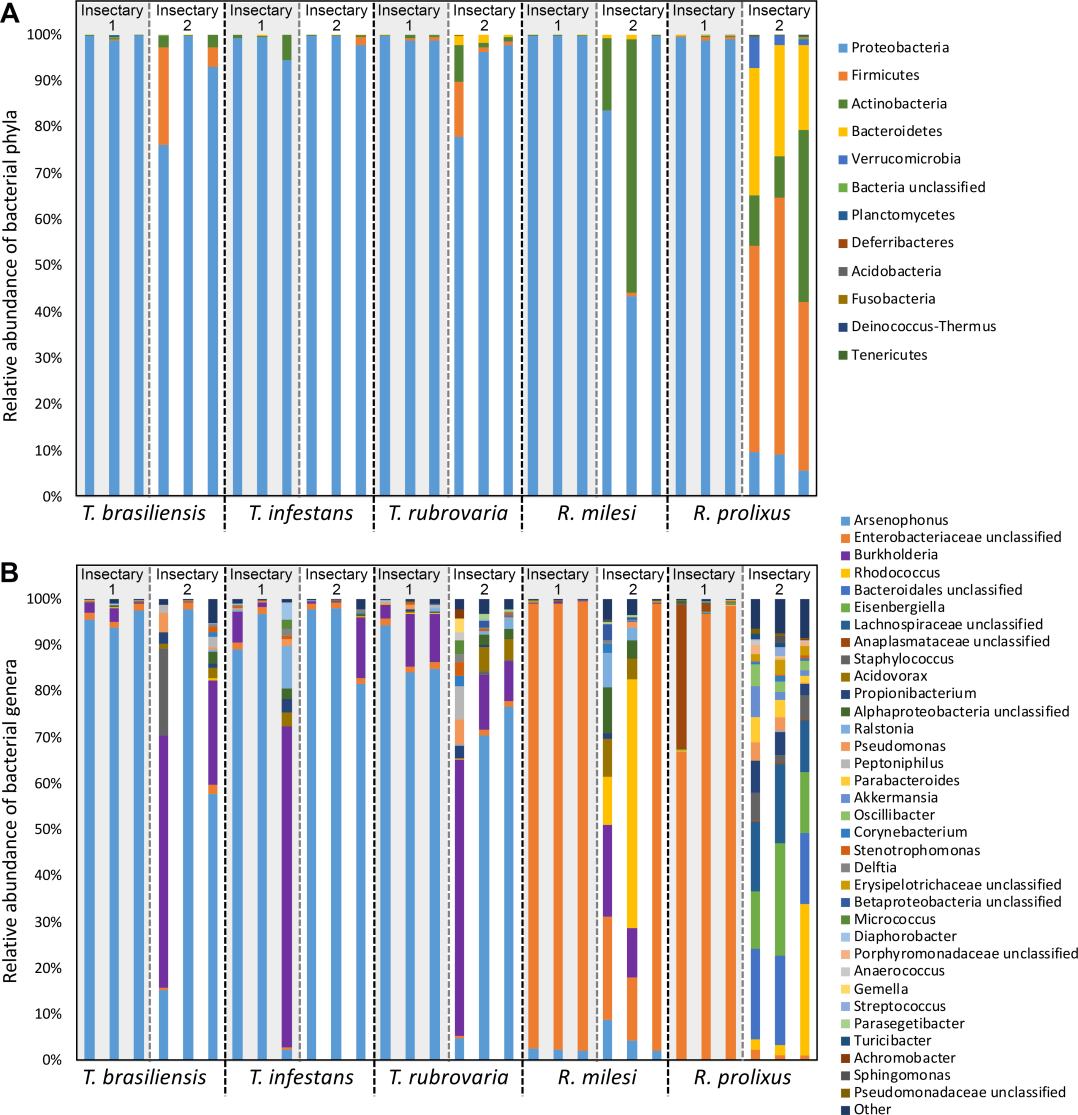
Taxonomic levels of microbiota composition from triatomine salivary glands. Taxonomic classification and relative percentage of the microbial community from *T*. *brasiliensis*, *T*. *infestans*, *T*. *rubrovaria*, *R*. *milesi* and *R*. *prolixus* salivary glands collected in Fiocruz (Insectary 1) and UFRJ (Insectary 2). The classification was based on RDP database using 80% of bootstrap cutoff. The relevant bacterial community was classified at phylum level **(A)** and genus level **(B**). Each bar represents a single triatomine.

The 16S rRNA sequences obtained from salivary glands were classified into 11 phyla. Most samples revealed high dominance of Proteobacteria (up to 99.9% of sequences), followed by Actinobacteria, Firmicutes and Bacteroidetes. However, the sequences obtained from *R*. *prolixus* collected in the UFRJ insectary exhibited the highest phylum diversity with predominance of Firmicutes (up to 40% of sequences) (Fig 2A). Taxonomic assignment at the genus level revealed the presence of 64 known genera along with 34 unclassified genera (classified into family, order or class). All genera accounting for at least 0.1% of the communities are graphically represented in Fig 2B.

Substantial differences in the bacterial community composition were observed among the insect genera. While the most abundant bacterial genus found in *Triatoma* spp. was *Arsenophus*, followed by *Burkholderia*, the most abundant bacteria in *Rhodnius* spp. belonged to unclassified Enterobacteriaceae family, followed by the *Rhodococcus* genus and the *Bacteriodailes* genus (Fig 2B).

Nonmetric multidimensional scaling was performed with Bray–Curtis distances to visualize the relationships among the bacterial community compositions based on the taxa present and their relative abundances, providing a score between 1 (complete similarity or no change) and 0 (complete dissimilarity or complete change).

**Fig 3:**
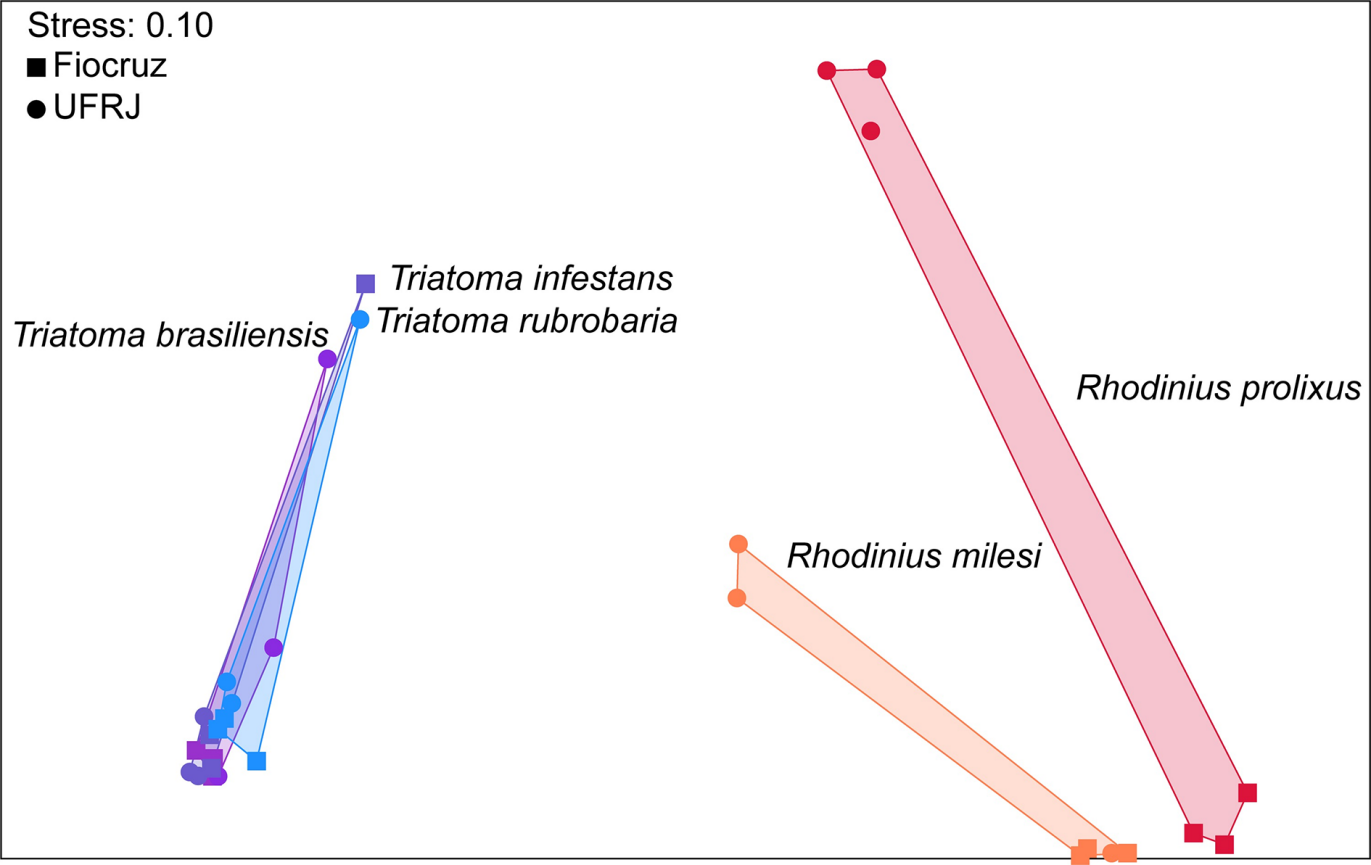
Nonmetric multidimensional scaling (NMDS) using Bray-Curtis dissimilarities based on OTU distribution of salivary-gland microbiota. Bacterial communities’ analysis based on Bray-Curtis distance from *T*. *infestans*, *T*. *brasiliensis*, *T*. *rubrovaria*, R. *prolixus* and *R*. *milesi* sequences collected in Fiocruz (Insectary 1) and UFRJ (Insectary.2).

Considering the ordination in the graph, the microbiomes were dispersed by the triatomine species as well as by the location sites. The *Triatoma* spp. bacterial communities were dispersed collectively on the left of the ordination, whereas the *Rhodnius* spp. bacterial communities were dispersed on the right (Fig 3). Moreover, the sites where the insects were kept influenced the microbial composition (Fig 3). The PERMANOVA analysis indicated highly significant effects of both insect genus and insectary (*p* < 0.01) as well as the interaction of the two factors (*p* < 0.01).

**Fig 4:**
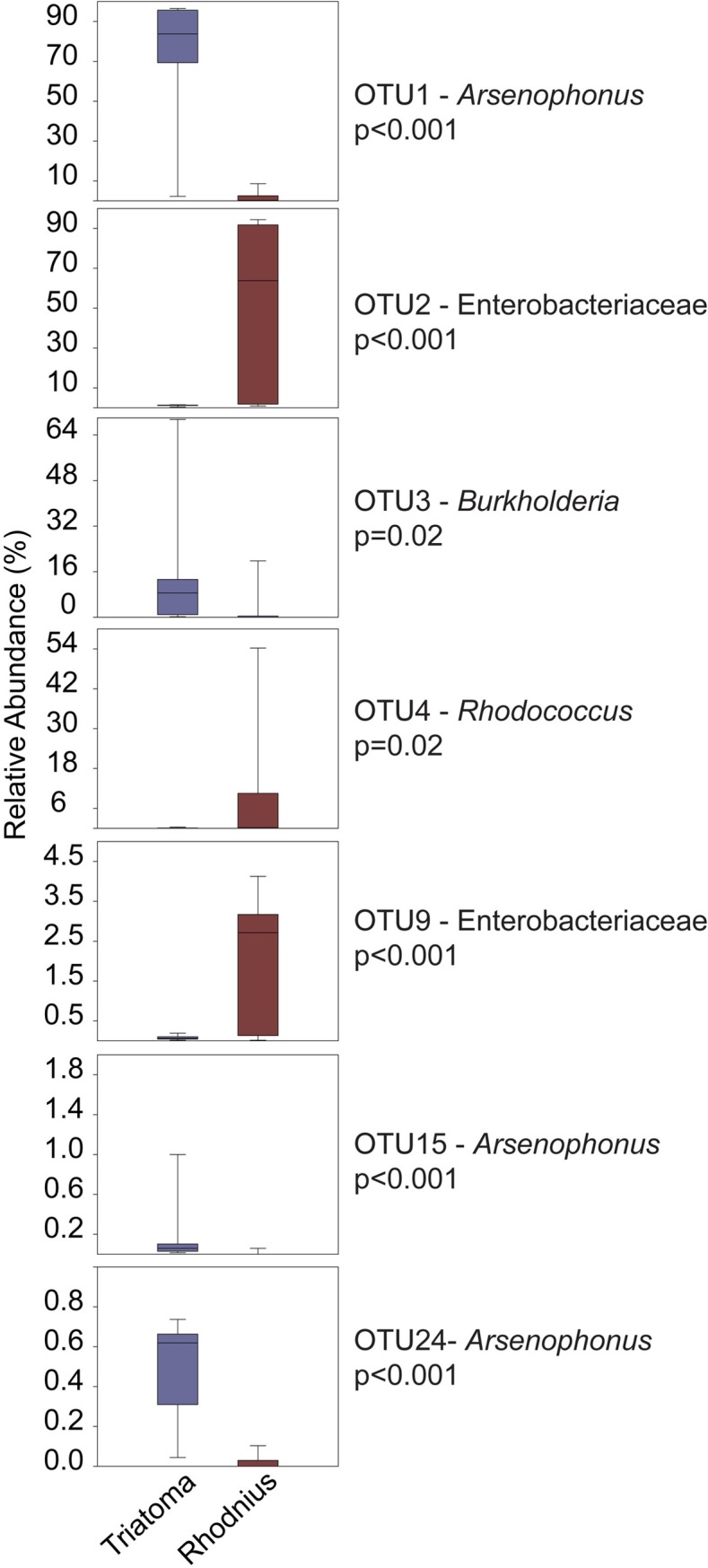
Principal OTUs responsible for observed differences in the salivary- gland microbiota. The principal bacterial OTUs from *T*. *brasiliensis*, *T*. *infestans*, *R*. *milesi*, *R*. *prolixus* and *T*. *rubrovaria* collected in UFRJ or Fiocruz insectary.

An indicator species analysis was performed to evaluate the relative abundances of the main bacterial OTUs between the two triatomine genera. Three OTUs were significantly more abundant in the *Triatoma* genus, two belonging to *Arsenophonus* bacterial genus and one belonging to *Stenotrophomonas* bacterial genus. Four OTUs were significantly more abundant in *Rhodnius*, two belonging to unclassified Enterobacteriaceae family, one to *Rhodococcus* genus and one to unclassified Betaproteobacteria class (Fig 4).

Only four OTUs were found in all samples: *Arsenophus*, *Rhodococcus*, and two units of unclassified Enterobacteriaceae. These units represent the core microbiome of the triatomine species analyzed in this study.

### Isolation of cultivable bacteria from vertebrate hosts

To investigate the influence of vertebrate hosts on the microbiota composition of salivary glands, cultivable bacteria were isolated from mouse back skin, human hand skin, and rabbit ear skin. A total of 22 CFUs were isolated from vertebrate hosts. Ten CFUs were isolated from rabbit skin, and two of them were found in the salivary glands of the triatomine species *Corynebacterium xerosis* and *R*. *rhodnii*. Eleven CFUs were isolated from human and mouse skin (Table 3).

**Table 3:** Bacterial isolates, their origin, growth medium and GenBank ID.

Source of isolation	Bacterial species	Identity (%)	Gen Bank acession #
**Rabbit**^**a**^	*Corynebacterium xerosis*	99.0	MF581327
**Rabbit**^**a**^	*Bacillus amyloliquefaciens*	100	MF581328
**Rabbit**^**a**^	*Staphylococcus saprophyticus*	100	MF581329
**Rabbit**^**a**^	*Jeotgalicoccus nanhaiensis*	100	MF581330
**Rabbit**^**a**^	*Rhodococcus rhodnii*	100	MF581331
**Rabbit**^**a**^	*Staphylococcus epidermidis*	100	MF581332
**Rabbit**^**a**^	*Rothia nasimurium*	99.0	MF581333
**Rabbit**^**a**^	*Staphylococcus* spp.	99.0	MF581334
**Rabbit**^**a**^	*Staphylococcus xylosus*	99.0	MF581336
**Rabbit**^**a**^	*Aerococcus viridans*	99.0	MF581337
**Human**^**a**^	*Micrococcus luteus*	100	MF581338
**Human**^**a**^	*Rothia nasimurium*	99.0	MF581318
**Human**^**a**^	*Pantoea septica*	99.0	MF581319
**Human**^**a**^	*Kocuria kristinae*	99.0	MF581320
**Human**^**a**^	*Staphylococcus xylosus*	99.0	MF581321
**Human**^**a**^	*Kytococcus sedentarius*	99.0	MF581323
**Human**^**a**^	*Staphylococcus epidermidis*	100	MF581324
**Human**^**a**^	*Bacillus niacini*	99.0	MF581325
**Human**^**a**^	*Staphyloccus xylosis*	99.0	MF581326
**Human**^**b**^	*Micrococcus luteus*	99.0	MF581317
**Human**^**b**^	*Staphylococcus* spp.	99.0	MF581335
**Mouse**^**b**^	*Staphylococcus* spp.	99.0	MG680926

a.Samples collect from UFRJ insectary

b. Samples collect from Instituto Oswaldo Cruz insectary

### Antagonistic interactions among bacterial isolates from triatomine salivary glands

**Fig 5:**
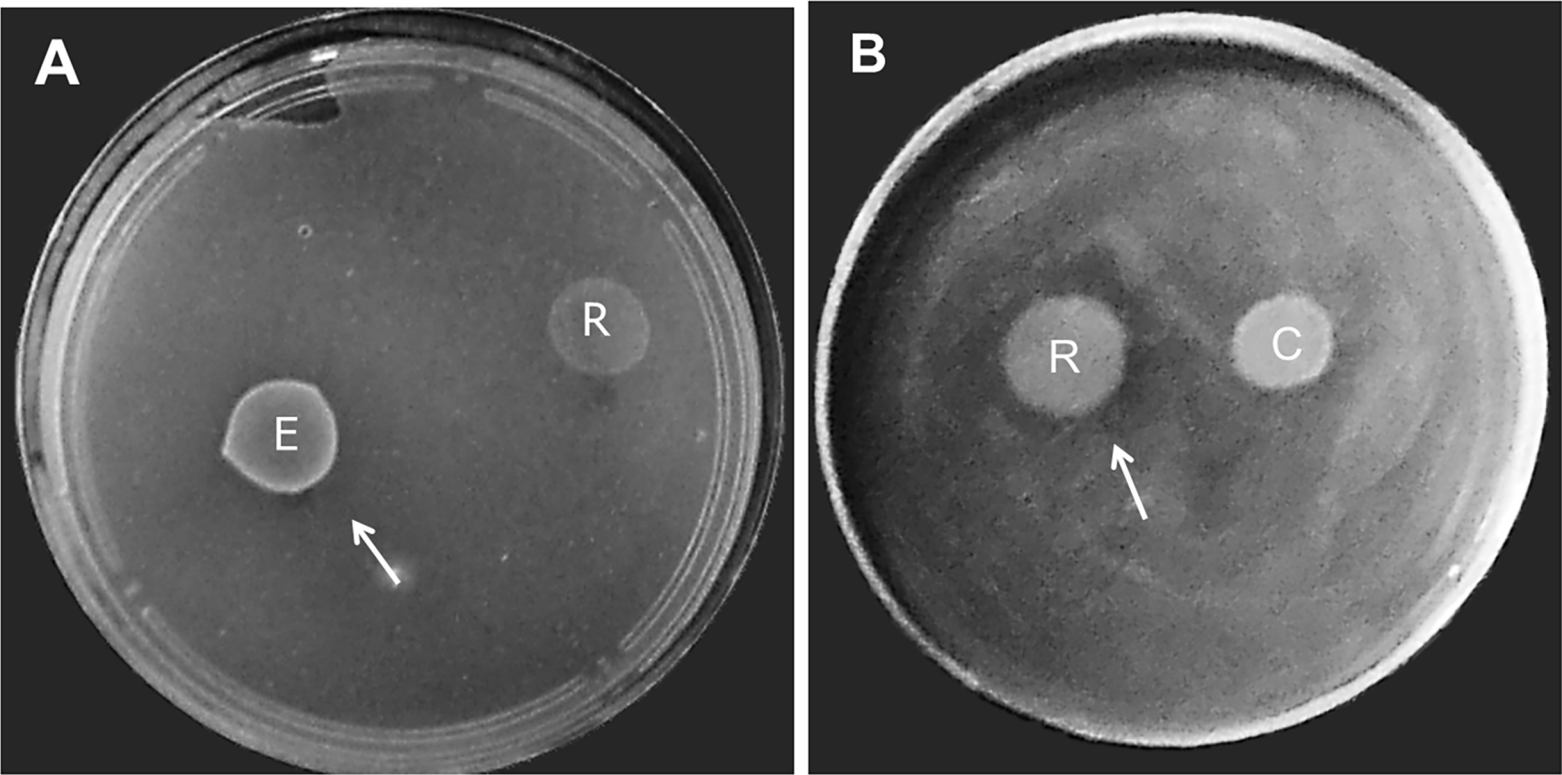
Antagonistic activity assay with bacterial isolates from triatomine salivary glands. Bacteria present in the saliva of *T*. *infestans* were isolated and subjected to antagonistic activity. **A-** *Corynebacterium xerosis* as indicator strain and **B-** *Enterococcus faecalis* were indicator strain. The producer strains were *Corynebacterium xerosis* (C); *Enterococcus faecalis* (E) and *Rhodococcus rhodnii* (R). The arrows indicate the inhibition halo.

To assess whether the cultivable bacteria present in the salivary glands were capable of producing substances that would inhibit the growth of other bacteria, the bacterial strains *P*. *mirabilis*, *C*. *xerosis*, *E*. *faecalis* and *R*. *rhodnii* isolated from triatomines were screened for antimicrobial substance production by cross-inhibition tests among the four strains. The results showed zones of inhibition larger than 8 mm in diameter. *E*. *faecalis* and *R*. *rhodnii* were the only bacteria exhibiting antagonistic activities against *C*. *xerosis* and *E*. *faecalis*, respectively (Fig 5 and S3 Fig).

The production of antimicrobial substances against *Escherichia coli* and *Staphylococcus aureus* was quantified in the four isolated strains, to assess whether the strains could inhibit bacteria of medical importance. The four strains had no antimicrobial activity against *S*. *aureus*. Only *E*. *faecalis* showed antimicrobial activity against *E*. *coli* (S4 Fig).

## Discussion

Insects are colonized by microorganisms which may offer fitness benefits: at least 15–20% of all insects live in symbiotic relationships with bacteria [25]. Bacterial communities can be influenced by conditions and resources in insect habitats, insect immunological tolerances and transmission mechanisms [3]. Microbial symbionts may contribute to the nutrition and development of insects and may produce bioactive compounds that protect the host against adverse conditions, predators and direct competitors, thereby increasing insect fitness [26, 27].

This study demonstrated the presence of different bacterial groups in the salivary glands of several triatomine species. In *Rhodnius* genus, the bacterium *R*. *rhodnii* was cultured from insects grown in the insectary, whereas *G*. *polyisoprenivorans* was cultured from insects (Table 2). *R*. *rhodnii* has been described as an endosymbiont present in the gut of *R*. *prolixus*. This species is important for insect development and insects that are not colonized by the endosymbiont reveal high mortality rates and can not molt [28, 29, 30, 31]. *T*. *infestans* harbored different culturable species of bacteria in its salivary glands (Table 2), suggesting that bacteria were acquired in the environment in which the insects were collected during the initial development stage of insects.

Certain bacteria identified in the salivary glands of triatomines (such as *R*. *rhodnii*, *E*. *faecalis* and *Corynebacterium* sp.) have also been observed in the guts of *P*. *megistus*, *R*. *prolixus* and *T*. *infestans*. Moreover, other bacteria were identified in different conditions and species, indicating the habitat conditions and the species may influence in the bacterial community establishment [11, 32]. Bacteria found in triatomines under laboratory conditions may not reflect natural characteristics, because among the wild *T*. *infestans*, 14 bacteria were isolated and these were not found in the same species kept in laboratory conditions [33]. Microbial composition differed among the triatomine species with *T*. *infestans* and *T*. *vitticeps* presenting the largest diversity of gut bacteria compared to *R*. *prolixus*, *P*. *megistus* and *Dipetalogaster maximus* [34].

To date most bacteria known in triatomines have been studied by culture methods. Bacteria isolated by these methods grow rapidly; however, they are rarely numerically dominant in the communities from which they are obtained. Culture methods may favor the growth of fast-growing bacteria at the expense of slow-growing species. It is estimated that 1% of the bacteria on Earth can be readily cultivated *in vitro*. Present estimates point to 61 distinct bacterial phyla, of which 31 reveal no cultivable representatives [35]. For endosymbionts, the ability to find cultivable representatives may be lower, since several bacterial symbionts depend on their hosts for their development. Therefore, we evaluated the bacterial communities from the salivary glands of *T*. *brasiliensis*, *T*. *infestans*, *T*. *rubrovaria*, *R*. *milesi* and *R*. *prolixus* with 16S rRNA gene sequencing.

The Shannon index was used to estimate the diversity, species count, and distribution of the bacterial species. In analyses of the bacterial species diversity, the triatomine salivary glands presented low diversity when compared with the bacterial community diversity in mammals, with few bacterial phyla dominating the communities (Figs 1, 2A and S2). Previous studies reveal different species of the genus *Anopheles* possess low bacterial diversity with few phylotypes with 16S rRNA sequencing [13]. In bees (*Apis mellifera*), 8–10 bacterial species constitute more than 98% of the gut bacterial community, indicating low bacterial diversity in the gut when compared to the other bacterial communities associated with the insects [36, 37, 38].

In insects, the establishment of bacterial communities depends on the bacterial tolerance toward unfavorable conditions such as pH, reactive nitrogen, oxygen species, concentration of antimicrobial compounds and host habit [39, 40, 41]; however, other factors may contribute to microbial richness and diversity, including microbial evolution, microbial biogeography, microbial dormancy, metabolic diversity and neutral processes [42].

The phylum Proteobacteria is highly diverse and contains a large variety of species that are adapted to several environments. For this reason, it was not a surprise to find this phylum with higher relative abundance in almost all bacterial communities found in the salivary glands of triatomines. This abundant phylum was also found in the salivary glands and guts of other insects [43, 44, 15]; however, dominance of the Firmicutes phylum as well as greater diversity and richness observed in the *R*. *prolixus* samples from the UFRJ insectary demonstrate the influence of environment on the bacterial composition.

In the UFRJ insectary, triatomines are in contact with different rabbits while feeding, increasing the chances of being colonized by several bacteria, culminating in higher diversity in salivary glands when compared with triatomines from Fiocruz. The increase in bacterial diversity found in *R*. *prolixus* obtained from the UFRJ insectary compared to *T*. *infestans*, *T*. *brasiliensis*, *T*. *rubrovaria* and *R*. *milesi* collected in the same insectary could be the result of an adaptation since the *R*. *prolixus* colony reared in the insectary for more than 30 years, suggesting the microbiota has already adapted to the environmental conditions, in contrast to other triatomine species, which have been established for less than 6 years (Fig 2). These results are corroborated in previous studies, which have revealed the biological and ecological factors such as age, genetics and environment can influence the composition of the bacterial communities [45].

According to our analysis of the microbial composition at the genus level, *Triatoma* spp are dominated by OTUs belonging to *Arsenophonus* bacterial genus, whereas the *Rhodnius* spp are dominated by an unclassified Enterobacteriaceae OTU. Thus, the relationships among bacteria and triatomines appear to be species-specific (Fig 2B). These species-specific relationships are also corroborated by the distribution of OTUs among the triatomine species (Figs 3 and 4). These similarities in the bacterial communities suggest the predominant bacterial OTUs differed among the triatomine genera. This hypothesis was based on the abundance of bacterial OTUs genera found in gut of certain insect species, such as bacterial OTU belonging to the *Serratia* spp. was dominant in *R*. *prolixus*, *Arsenophonus* spp. OTU in *T*. *infestans* and in *P*. *megistus* and *Candidatus Rohrkolberia cinguli* OTU in *D*. *maximus* [34].

To evaluate the influence of vertebrate host on the bacterial composition of salivary glands, a total of 22 CFUs were isolated from rabbit, human and mouse skin from the UFRJ and Fiocruz insectaries. The majority of bacteria identified such as *Bacillus amyloliquefaciens*, *Pantoea* sp., *R*. *rhodnii* and *Micrococcus luteus* are frequently present in the soil and water, whereas others such as *Staphylococcus saprophyticus*, *C*. *xerosis* and *S*. *epidermidis* are found in humans (Table 3). In humans, these bacteria are opportunistic and are found on the skin and in the urinary tract, causing urinary tract infections and hospitalization [46].

It may be that, the bacterial strains present in the insects were acquired through contact with hosts, because bacterial species (*C*. *xerosis* and *R*. *rhodnii*) present on the vertebrate hosts were found in the salivary glands, suggesting horizontal transmission. Indeed bacteria can be acquired by more than one route such as trophalaxia, crophophagy and vertical or transovarian transmission [47, 48, 49]. In *R*. *prolixus*, *R*. *rhodnii* transmission occurred by coprophagy [50]; however, in *T*. *infestans*, vertical transovarial transmission occurs, due to the presence of bacteria in the embryonic gut prior to the egg hatching [51].

Most bacterial isolates studied in this work did not reveal antagonistic activities against each other or against the indicator species, with the exception of *E*. *faecalis*, which was able to inhibit *C*. *xerosis* and *E*. *coli*; a Gram-positive bacterium (*R*. *rhodnii)* also inhibited *E*. *faecalis*, suggesting interbacterial modulation, if present, occurs in a species-specific manner (Figs 5, S3 and S4).

The gut microbiota can be controlled by the IMD immunological pathway via lysozymes and antimicrobial peptides, resulting in a decrease in the number of bacterial species a few days after feeding [52, 53]. In contrast, when triatomines are infected with *T*. *cruzi* the microbial composition changes in a species-specific manner. This parasite modulates the vector immunity, increasing the basal response against microbial proliferation such as the prophenoloxidase complex, antimicrobial proteases, and antimicrobial peptides (AMPs). The competition between the parasite and microbiota will determine the success of colonization by trypanosomes in the insect gut [54, 55, 56, 2].

A strategy to eliminate Chagas disease is paratransgenesis, where the symbiotic bacteria are isolated and genetically transformed *in vitro*. The symbiont is altered and reintroduced into the host vector to produce molecules that interfere with the pathogen transmission [57]. In *R*. *prolixus*, the genetic modification of an endosymbiotic bacterium, *R*. *rhodnii* produced an antiparasitic peptide called cecropin A. In the gut infected with *T*. *cruzi*, cecropin A was able to eliminate 65% of the parasites. Thus a genetically altered symbiont could be used to eliminate the transport of an infectious agent from a vector [58, 59]. Therefore, it is essential to understand the microbial composition and its relationship with the insects in order to select adequate bacterial strain for paratransgenesis.

In our study, we fully characterized bacterial communities in the salivary glands of triatomines. Bacterial communities varied among triatomine species and were species-specific. As our molecular-level understanding of the influence of bacteria on vector competence remains limited, further studies are needed to understand whether the microbiota is capable of interfering with *T*. *cruzi* colonization in insects.

## Supporting information

S1 Fig Map of South America indicating the origin of triatomines. Geographic locations of triatomines collected in South America. **A-** Campo de Santana do Mato; **B-** Novo Horizonte; **C-** Bambuí; **D-** Belo Horizonte; **E-** UFRJ insectary; **F-** Fiocruz insectary; **G-** Santa Rosa and **H-** Chaco.
(TIF)

S2 Fig Rarefaction curves of 16S rRNA sequences from triatomines salivary glands microbiota. The number of different bacterial species is given as a function of the number of sequences obtained by Illumina sequencing. Each colored line represents the OTUs from *T*. *brasiliensis* (Red), *T*. *infestans* (Blue), *R*. *milesi* (Yellow), *R*. *prolixus* (Green) and *T*. *rubrovaria* (Purple) collected in UFRJ or Fiocruz insectary.
(TIF)

S3 Fig Antagonistic activity assay with bacterial isolates from triatomine salivary glands. Bacteria present in the saliva of the *T*. *infestans* were isolated and subjected to antagonistic activity. **A-** *Proteus mirabilis* were indicator strain; **B-***Corynebacterium xerosis* as indicator strain. **C-** *Enterococcus faecalis* as indicator strain. **D-** and **E-** *Rhodococcus rhodnii* as indicator strain. The producer strains were *Proteus mirabilis* (P); *Corynebacterium xerosis* (C); *Enterococcus faecalis* (E).
(TIF)

S4 Fig Antagonistic activity assay with bacterial isolates from triatomine salivary glands. Bacteria present in the saliva of the *T*. *infestans* were isolated and subjected to antagonistic activity. **A** and **E-** *Proteus mirabilis* as producer strain; **B** and **F-** *Corynebacterium xerosis* as producer strains; **C** and **G-** *Enterococcus faecalis* as producer strain; **D** and **H-** *Rhodococcus rhodnii* as producer strain. The *Escherichia coli* ATCC 25922 in the first line and *Staphylococcus aureus* ATCC 29213 in the second line were used as indicator strain. The arrows indicate the inhibition halo.
(TIF)

S1 Table Samples summary, in relation to genera, insectary of origin, richness and diversity indexes and number of sequences, before and after quality check. (TIF)

## References

[1] Ramirez JL, Souza-Neto J, Cosme RT, Rovira J, Ortiz A, Pascale JM, et al (2012). Reciprocal tripartite interactions between the *Aedes aegypti* midgut microbiota, innate immune system and dengue virus influences vector competence. PLoS Neglected Tropical Diseases.6: e1561 10.1371/journal.pntd.0001561 . 22413032 PMC3295821

[2] Díaz S, Villavicencio B, Correia N, Costa J, Haag KL. (2016). Triatomine bugs, their microbiota and *Trypanosoma cruzi*: asymmetric responses of bacteria to an infected blood meal. Parasites & Vectors. 9: 636 10.1186/s13071-016-1926-2 . 27938415 PMC5148865

[3] Douglas AE. (2015). Multiorganismal insects: diversity and function of resident microorganisms. Annual Review of Entomology.60: 17–34. 10.1146/annurev-ento-010814-020822 . 25341109 PMC4465791

[4] Dillon RJ and Dillon VM. (2004). The gut bacteria of insects: nonpathogenic interactions. Annual Review of Entomology. 49: 71–92. 10.1146/annurev.ento.49.061802.123416 . 14651457

[5] Garcia ES, Castro DP, Figueiredo MB, Azambuja P. (2010). Immune homeostasis to microorganisms in the guts of triatomines (Reduviidae)—A review. Memorias do Instituto Oswaldo Cruz. 105: 605–610. 10.1590/S0074-02762010000500001 . 20835604

[6] Duguma D, Hall MW, Rugman-Jones P, Stouthamer R, Terenius O, Neufeld JD, et al(2015). Developmental succession of the microbiome of *Culex* mosquitoes Ecological and evolutionary microbiology. BMC Microbiology. 15: 140 10.1186/s12866-015-0475-8 . 26205080 PMC4513620

[7] Buck M, Nilsson LKJ, Brunius C, Dabiré RK, Hopkins R, Terenius O. (2016). Bacterial associations reveal spatial population dynamics in *Anopheles gambiae* mosquitoes. Scientific Reports. Nature Publishing Group. 6: 1–9. 10.1038/s41598-016-0001-8 . 26960555 PMC4785398

[8] Muturi EJ, Kim CH, Bara J, Bach EM, Siddappaji MH. (2016). *Culex pipiens* and *Culex restuans* mosquitoes harbor distinct microbiota dominated by few bacterial taxa. Parasites & Vectors. 9:18 10.1186/s13071-016-1299-6 . 26762514 PMC4712599

[9] Rani A, Sharma A, Rajagopal R, Adak T, Bhatnagar RK. (2009). Bacterial diversity analysis of larvae and adult midgut microflora using culture-dependent and culture-independent methods in lab-reared and field-collected *Anopheles stephensi—*an Asian malarial vector. BMC Microbiology. 9:96 10.1186/1471-2180-9-96 . 19450290 PMC2698833

[10] Osei-Poku J, Mbogo CM, Palmer WJ, Jiggins FM. (2012). Deep sequencing reveals extensive variation in the gut microbiota of wild mosquitoes from Kenya. Molecular Ecology. 21: 5138–5150. 10.1111/j.1365-294X.2012.05759.x . 22988916

[11] Vallejo GA, Guhl F, Schaub GA. (2009). Triatominae-*Trypanosoma cruzi/T*. *rangeli*: vector-parasite interactions. Acta Tropica. 110: 137–147. 10.1016/j.actatropica.2008.10.001 . 18992212

[12] Vieira CS, Mattos DP, Waniek PJ, Santangelo JM, Figueiredo MB, Gumiel M, et al (2015). *Rhodnius prolixus* interaction with *Trypanosoma rangeli*: modulation of the immune system and microbiota population. Parasites & Vectors. 8: 135 10.1186/s13071-015-0736-2 . 25888720 PMC4350287

[13] Favia G, Ricci I, Damiani C, Raddadi N, Crotti E, Marzorati M, et al (2007). Bacteria of the genus *Asaia* stably associate with *Anopheles stephensi*, an Asian malarial mosquito vector. Proceedings of the National Academy of Sciences. 104: 9047–9051. 10.1073/pnas.0610451104 . 17502606 PMC1885625

[14] Qiu Y, Nakao R, Ohnuma A, Kawamori F, Sugimoto C. (2014). Microbial population analysis of the salivary glands of ticks; a possible strategy for the surveillance of bacterial pathogens. PLoS ONE. 9: e103961 10.1371/journal.pone.0103961 . 25089898 PMC4121176

[15] Sharma P, Sharma S, Maurya RK, Das De T, Thomas T, Lata S, et al (2014). Salivary glands harbor more diverse microbial communities than gut in *Anopheles culicifacies*. Parasites & Vectors. 7: 235 10.1186/1756-3305-7-235 . 24886293 PMC4062515

[16] Weisburg WG, Barns SM, Pelletier DA, Lane DJ. (1991). 16S Ribosomal DNA amplification for phylogenetic study. Journal of Bacteriology.173: 697–703. . 1987160 10.1128/jb.173.2.697-703.1991 PMC207061

[17] Pruesse E, Quast C, Knittel K, Fuchs BM, Ludwig W, Peplies J, et al (2007). SILVA: A comprehensive online resource for quality checked and aligned ribosomal RNA sequence data compatible with ARB. Nucleic Acids Research. 35: 7188–7196. 10.1093/nar/gkm864 . 17947321 PMC2175337

[18] Paulson AR, Von Aderkas P, Perlman SJ. (2014). Bacterial associates of seed-parasitic wasps (Hymenoptera: Megastigmus). BMC Microbiology. 14: 224 10.1186/s12866-014-0224-4 . 25286971 PMC4197294

[19] Schloss PD, Westcott SL, Ryabin T, Hall JR, Hartmann M, Hollister EB, et al (2009). Introducing mothur: open-source, platform-independent, community-supported software for describing and comparing microbial communities. Applied and Environmental Microbiology. 75: 7537–7541. 10.1128/AEM.01541-09 . 19801464 PMC2786419

[20] Quast C, Pruesse E, Yilmaz P, Gerken J, Schweer T, Yarza P, et al (2013). The SILVA ribosomal RNA gene database project: improved data processing and web-based tools. Nucleic Acids Research. 41: 590–596. 10.1093/nar/gks1219 . 23193283 PMC3531112

[21] Cole JR, Wang Q, Cardenas E, Fish J, Chai B, Farris RJ, et al (2009). The ribosomal database project: improved alignments and new tools for rRNA analysis. Nucleic Acids Research. 37: 141–145. 10.1093/nar/gkn879 . 19004872 PMC2686447

[22] Hammer, Harper D. A. T., & Ryan P. D. (2001). PAST: Paleontological statistics software package for education and data analysis. Palaeontologia Electronica. 4: 1–9. Available from: http://palaeo-electronica.org/2001_1/past/issue1_01.htm.

[23] Dufrêne M, Legendre P. (1997). Species assemblages and indicator species: the need for a flexible asymmetrical approach. Ecological Monographs. 67: 345–366. 10.2307/2963459 PMID: 1344.

[24] Marinho PR, Moreira APB, Pellegrino FLPC, Muricy G, Bastos M do C de F, dos Santos KRN, et al (2009). Marine *Pseudomonas putida*: a potential source of antimicrobial substances against antibiotic-resistant bacteria. Memorias do Instituto Oswaldo Cruz. 104: 678–682. 10.1590/S0074-02762009000500002 . 19820824

[25] Buchner P. (1965). Endosymbiosis of animals with plant microorganisms. Mycologia 60:466 10.2307/3757184

[26] Brownlie JC, Johnson KN. (2009). Symbiont-mediated protection in insect hosts. Trends in Microbiology. 17: 348–354. 10.1016/j.tim.2009.05.005 . 19660955

[27] Chaves S, Neto M, Tenreiro R. (2009). Insect-symbiont systems: From complex relationships to biotechnological applications. Biotechnology Journal. 4: 1753–1765. 10.1002/biot.200800237 . 19844913

[28] Harington J. (1960). Studies on *Rhodnius prolixus*: growth and development of normal and sterile bugs, and the symbiotic relationship. Parasitology. 50: 279–286. 10.1017/S0031182000025373 . 14399809

[29] Nyirady SA. (1973). The germfree culture of three species of Triatominae: *Triatoma protracta*, *Triatoma rubida* and *Rhodnius prolixus* Stal. Journal of Medical Entomology. 10: 417–48. . 4586544 10.1093/jmedent/10.5.417

[30] Hill P, Campbell JA, Petrie IA. (1976). *Rhodnius prolixus* and its symbiotic actinomycete: a microbiological, physiological and behavioural study. Proceedings of the Royal Society B: Biological Sciences. 194: 501–525. 10.1098/rspb.1976.0091 PMID: 12514. 12514

[31] Dasch GA, Weiss E, Chang K. (1984). Endosymbionts of insects. In Bergey’s Manual of Systematic Bacteriology. 811–33.

[32] Gumiel M, Da Mota FF, Rizzo VDS, Sarquis O, Castro DP De, Lima MM, et al (2015). Characterization of the microbiota in the guts of *Triatoma brasiliensis* and *Triatoma pseudomaculata* infected by *Trypanosoma cruzi* in natural conditions using culture independent methods. Parasites & Vectors. 8: 245 10.1186/s13071-015-0836-z . 25903360 PMC4429471

[33] Eichler S., Reintjes N., Jung M., Yassin A.F., Schaal K.P., Junqueira A., et al (1996). Identification of bacterial isolates and symbionts from wild populations of *Triatoma infestans* and *Triatoma sordida*. Memórias do Instituto Oswaldo Cruz. 91 (Suppl.):125.

[34] da Mota FF, Marinho LP, de Moreira CJC, Lima MM, Mello CB, Garcia ES, et al (2012). Cultivation-independent methods reveal differences among bacterial gut microbiota in triatomine vectors of Chagas disease. PLoS Neglected Tropical Diseases. 6:e1631 10.1371/journal.pntd.0001631 . 22563511 PMC3341335

[35] Vartoukian SR, Palmer RM, Wade WG. (2010). Strategies for culture of ‘unculturable’ bacteria. FEMS Microbiology Letters. 309: 1–7. 10.1111/j.1574-6968.2010.02000.x . 20487025

[36] Ahn JH, Hong IP, Bok JI, Kim BY, Song J, and Weon HY. (2012). Pyrosequencing analysis of the bacterial communities in the guts of honey bees. *Apiscerana* and *Apismellifera* in Korea. The Journal of Microbiology. 50: 735–745. 10.1007/s12275-012-2188-0 . 23124740

[37] Moran NA, Hansen AK, Powell JE, Sabree ZL. (2012). Distinctive gut microbiota of honey bees assessed using deep sampling from individual worker bees. PLoS ONE. 7: e36393 10.1371/journal.pone.0036393 . 22558460 PMC3338667

[38] Corby-Harris V, Maes P, Anderson KE. (2014). The bacterial communities associated with honey bee (*Apis mellifera)* foragers. PLoS ONE. 9 e95056 10.1371/journal.pone.0095056 . 24740297 PMC3989306

[39] Bezerra CM, de Góes Cavalcanti LP, de Souza R de CM, Barbosa SE, Xavier SC das C, Jansen AM, et al (2014). Domestic, Peridomestic and wild hosts in the transmission of *Trypanosoma cruzi* in the caatinga area colonised by *Triatoma brasiliensis*. Memorias do Instituto Oswaldo Cruz. 109: 887–898. 10.1590/0074-0276140048 . 25410992 PMC4296493

[40] Donaldson GP, Lee SM, Mazmanian SK. (2015). Gut biogeography of the bacterial microbiota. Nature Reviews Microbiology. 14: 20–32. 10.1038/nrmicro3552 . 26499895 PMC4837114

[41] Whitten M, Sun F, Tew I, Schaub G, Soukou C, Nappi A, et al (2007). Differential modulation of *Rhodnius prolixus* nitric oxide activities following challenge with *Trypanosoma rangeli*, *T*. *cruzi* and bacterial cell wall components. Insect Biochemistry and Molecular Biology. 37: 440–452. 10.1016/j.ibmb.2007.02.001 . 17456439

[42] Fierer N, Lennon JT. (2011). The generation and maintenance of diversity in microbial communities. American Journal of Botany. 98: 439–448. 10.3732/ajb.1000498 . 21613137

[43] Wang Y, Gilbreath TM, Kukutla P, Yan G, Xu J. (2011). Dynamic gut microbiome across life history of the malaria mosquito *Anopheles gambiae* in Kenya. PLoS ONE. 6: e24767 10.1371/journal.pone.0024767 . 21957459 PMC3177825

[44] Chandel K, Mendki MJ, Parikh RY, Kulkarni G, Tikar SN, Sukumaran D, et al (2013). Midgut microbial community of *Culex quinquefasciatus* mosquito populations from India. PLoS ONE. 8 e80453 10.1371/journal.pone.0080453 . 24312223 PMC3843677

[45] Pernice M, Simpson SJ, Ponton F. (2014). Towards an integrated understanding of gut microbiota using insects as model systems. Journal of Insect Physiology. 69: 12–18. 10.1016/j.jinsphys.2014.05.016 . 24862156

[46] Grice EA. (2013). The skin microbiome. Nature Reviews Microbiology. 9: 244–253. 10.1038/nrmicro2537 . 21407241 PMC3535073

[47] Coyte KZ, Schluter J, Foster KR. (2015). The ecology of the microbiome: Networks, competition, and stability. Science. 350: 663–666. 10.1126/science.aad2602 . 26542567

[48] Devevey G, Dang T, Graves CJ, Murray S, Brisson D. (2015). First arrived takes all: Inhibitory priority effects dominate competition between co-infecting *Borrelia burgdorferi* strains. Ecological and evolutionary microbiology. BMC Microbiology. 15:61 10.1186/s12866-015-0381-0 25887119 PMC4359528

[49] Wang Y, Rozen DE. (2017). Gut microbiota colonization and *Nicrophorus vespilloides* throughout development. Applied and Environmental Microbiology. 83: 1–13. 10.1128/AEM.03250-16 . 28213538 PMC5394326

[50] Brecher G, Wigglesworth VB. (1944). The transmission of *Actinomyces rhodnii* in *Rhodnius prolixus* Stal (hemiptera) and its influence on the growth of the host. Parasitology. 35: 220–224. 10.1017/S0031182000021648

[51] Hypsa V. (1993). Endocytobionts of *Triatoma infestans*—distribution and transmission. Journal of Invertebrate Pathology. 61: 32–38. 10.1006/jipa.1993.1006

[52] Azambuja P, Feder D, Garcia ES. (2004). Isolation of *Serratia marcescens* in the midgut of *Rhodnius prolixus*: impact on the establishment of the parasite *Trypanosoma cruzi* in the vector. Experimental Parasitology. 107: 89–96. 10.1016/j.exppara.2004.04.007 . 15208042

[53] Garcia ES, Castro DP, Figueiredo MB, Azambuja P. (2010). Immune homeostasis to microorganisms in the guts of triatomines (Reduviidae)—a review. Memorias do Instituto Oswaldo Cruz.105: 605–610. 10.1590/S0074-02762010000500001 . 20835604

[54] Castro DP, Moraes CS, Gonzalez MS, Ratcliffe NA, Azambuja P, Garcia ES. (2012). *Trypanosoma cruzi* immune response modulation decreases microbiota in *Rhodnius prolixus* gut and is crucial for parasite survival and development. PLoS ONE. 7: e36591 10.1371/journal.pone.0036591 . 22574189 PMC3344921

[55] Soares TS, Buarque DS, Queiroz BR, Gomes CM, Braz GRC, Araújo RN, et al (2015). A kazal-type inhibitor is modulated by *Trypanosoma cruzi* to control microbiota inside the anterior midgut of *Rhodnius prolixus*. Biochimie. 112: 41–48. 10.1016/j.biochi.2015.02.014 . 25731714

[56] Vieira C, Waniek P, Castro D, Mattos D, Moreira O, Azambuja P. (2016). Impact of *Trypanosoma cruzi* on antimicrobial peptide gene expression and activity in the fat body and midgut of *Rhodnius prolixus*. Parasites & Vectors. 9: 119 10.1186/s13071-016-1398-4 . 26931761 PMC4774030

[57] Hurwitz I, Fieck A, Read A, Hillesland H, Klein N, Kang A, et al (2011). Paratransgenic control of vector borne diseases. International Journal of Biological Sciences. 7: 1334–1344. 10.7150/ijbs.7.1334 . 22110385 PMC3221369

[58] Beard CB, Durvasula RV, Richards FF. (2000). Bacterial symbiont transformation in Chagas disease vectors. Insect Transgenesis. 289–303. 10.1201/9781420039399.ch16

[59] Durvasula R V., Gumbs A, Panackal A, Kruglov O, Aksoy S, Merrifield RB, et al (1997). Prevention of insect-borne disease: an approach using transgenic symbiotic bacteria. Proceedings of the National Academy of Sciences. 94: 3274–3278. 10.1073/pnas.94.7.3274 . 9096383 PMC20359

