# Supplementary figures and images for: Bacterial community composition in the salivary glands of triatomines (Hemiptera: Reduviidae)
Source: PLoS Negl Trop Dis. 2018 Sep 13;12(9):e0006739. doi: 10.1371/journal.pntd.0006739 (PMC6136693; doi:10.1371/journal.pntd.0006739)

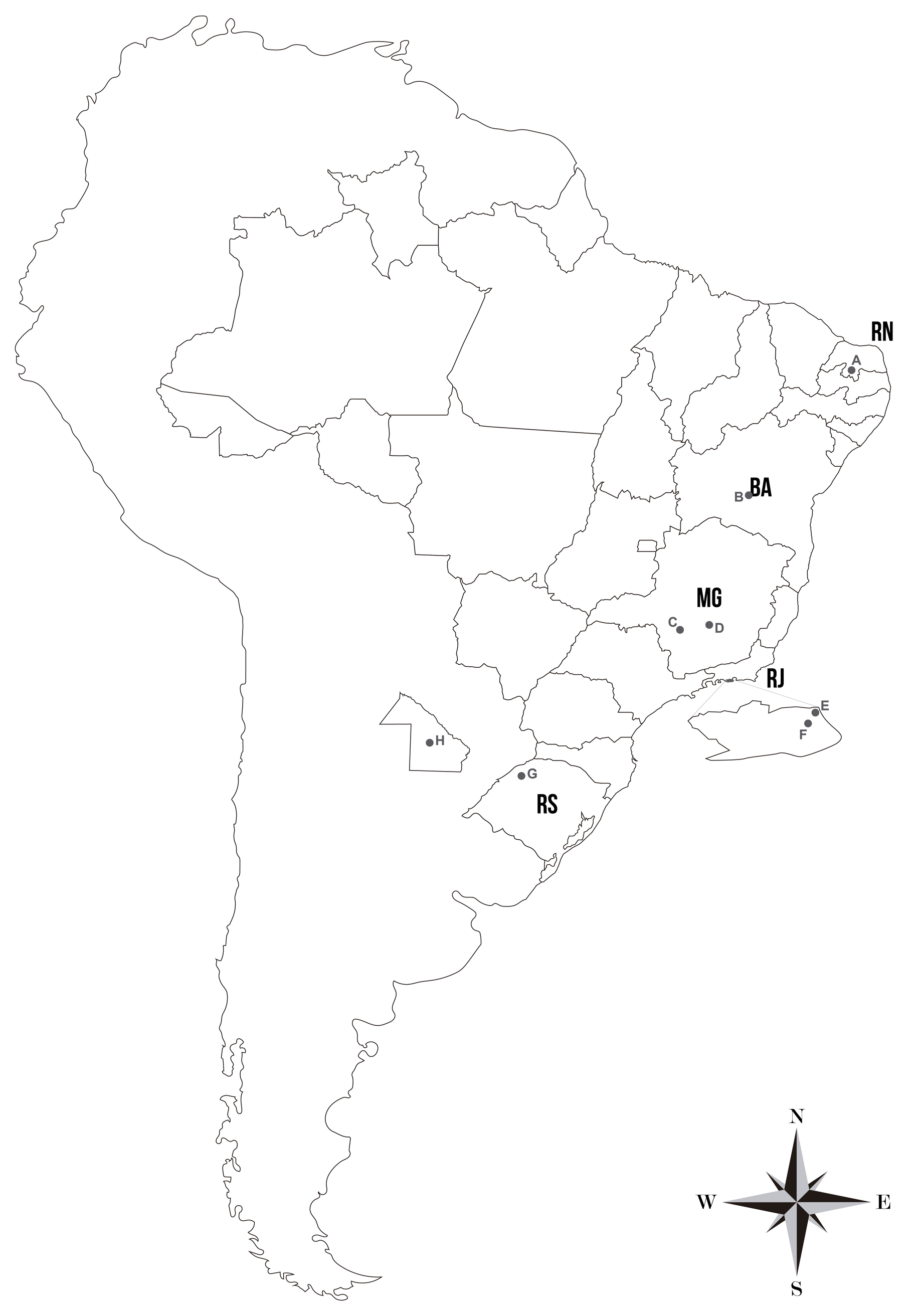

Supplement: S1 Fig — Geographic locations of triatomines collected in South America. A- Campo de Santana do Mato; B- Novo Horizonte; C- Bambuí; D- Belo Horizonte; E- UFRJ insectary; F- Fiocruz insectary; G- Santa Rosa and H- Chaco. (TIF) [file pntd.0006739.s001.tif]

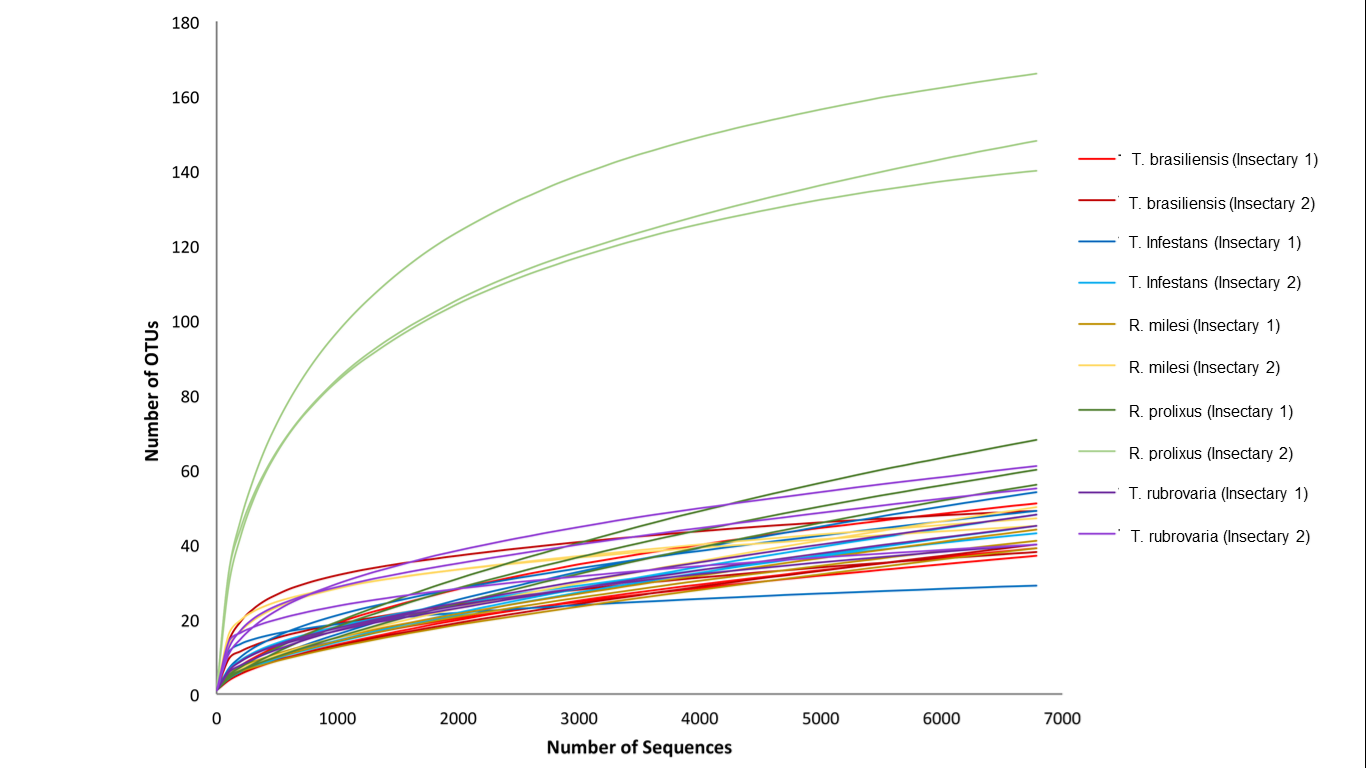

Supplement: S2 Fig — The number of different bacterial species is given as a function of the number of sequences obtained by Illumina sequencing. Each colored line represents the OTUs from T. brasiliensis (Red), T. infestans (Blue), R. milesi (Yellow), R. prolixus (Green) and T. rubrovaria (Purple) collected in UFRJ or Fiocruz insectary. (TIF) [file pntd.0006739.s002.tif]

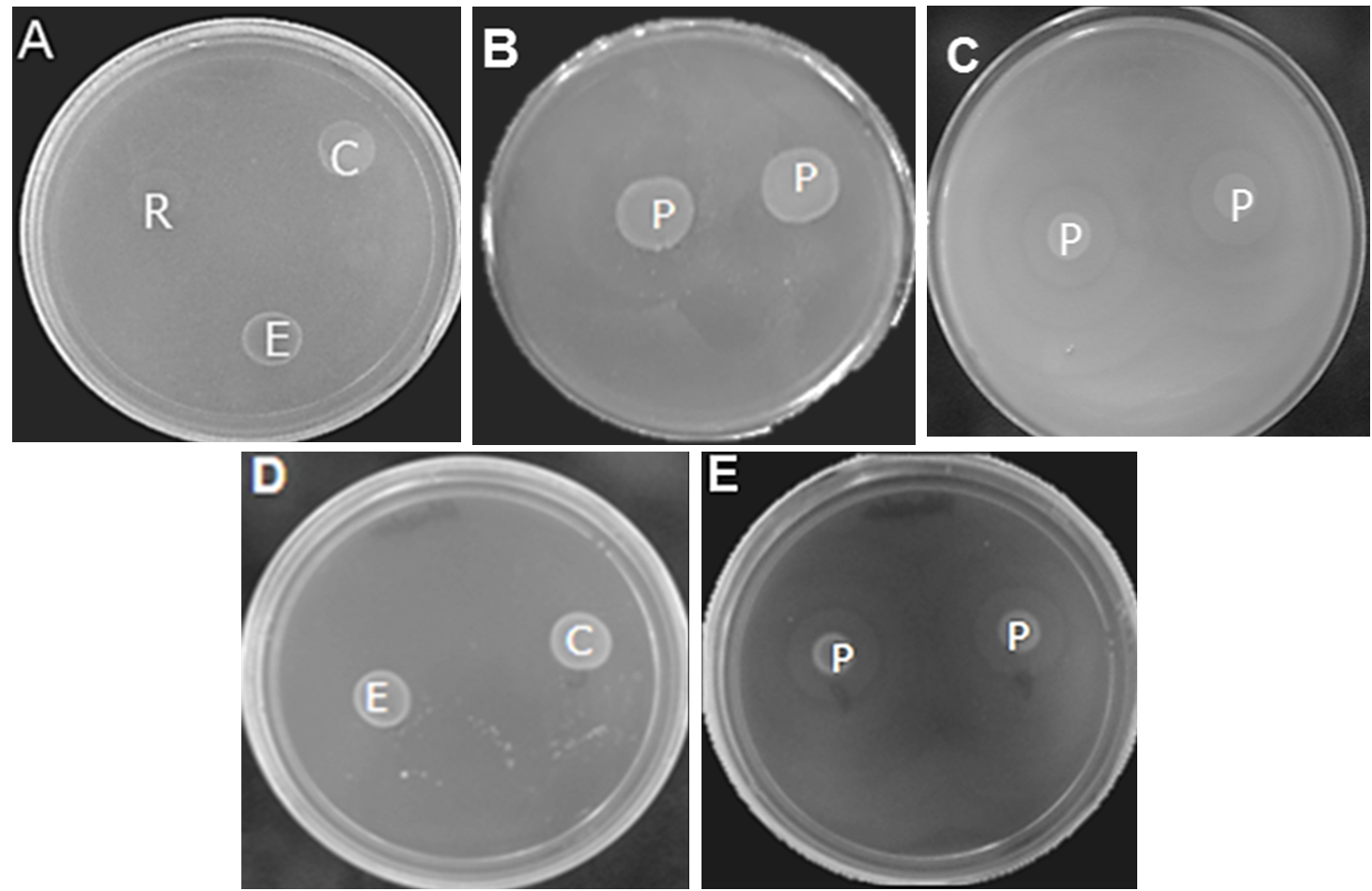

Supplement: S3 Fig — Bacteria present in the saliva of the T. infestans were isolated and subjected to antagonistic activity. A- Proteus mirabilis were indicator strain; B-Corynebacterium xerosis as indicator strain. C- Enterococcus faecalis as indicator strain. D- and E- Rhodococcus rhodnii as indicator strain. The producer strains were Proteus mirabilis (P); Corynebacterium xerosis (C); Enterococcus faecalis (E). (TIF) [file pntd.0006739.s003.tif]

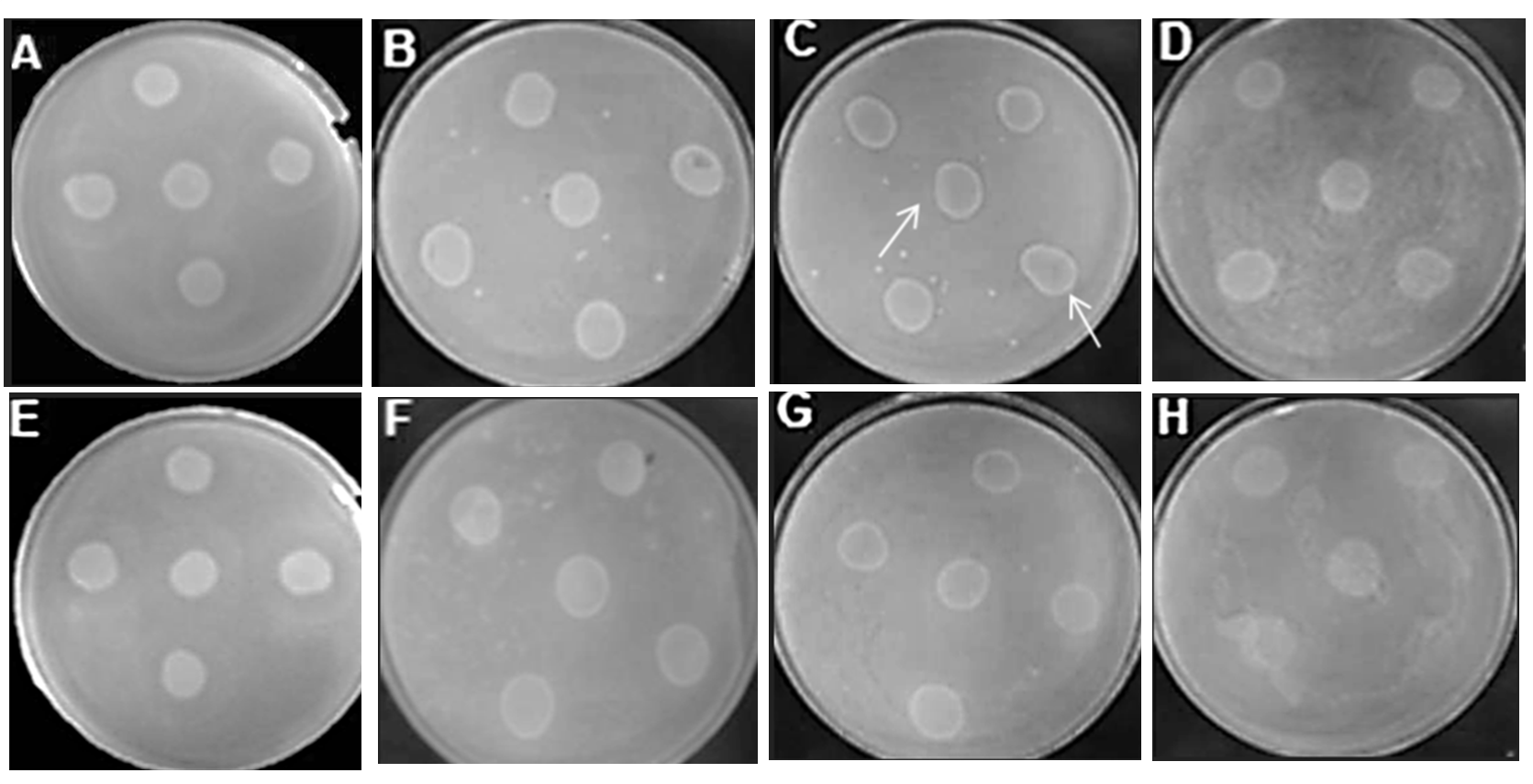

Supplement: S4 Fig — Bacteria present in the saliva of the T. infestans were isolated and subjected to antagonistic activity. A and E- Proteus mirabilis as producer strain; B and F- Corynebacterium xerosis as producer strains; C and G- Enterococcus faecalis as producer strain; D and H- Rhodococcus rhodnii as producer strain. The Escherichia coli ATCC 25922 in the first line and Staphylococcus aureus ATCC 29213 in the second line were used as indicator strain. The arrows indicate the inhibition halo. (TIF) [file pntd.0006739.s004.tif]

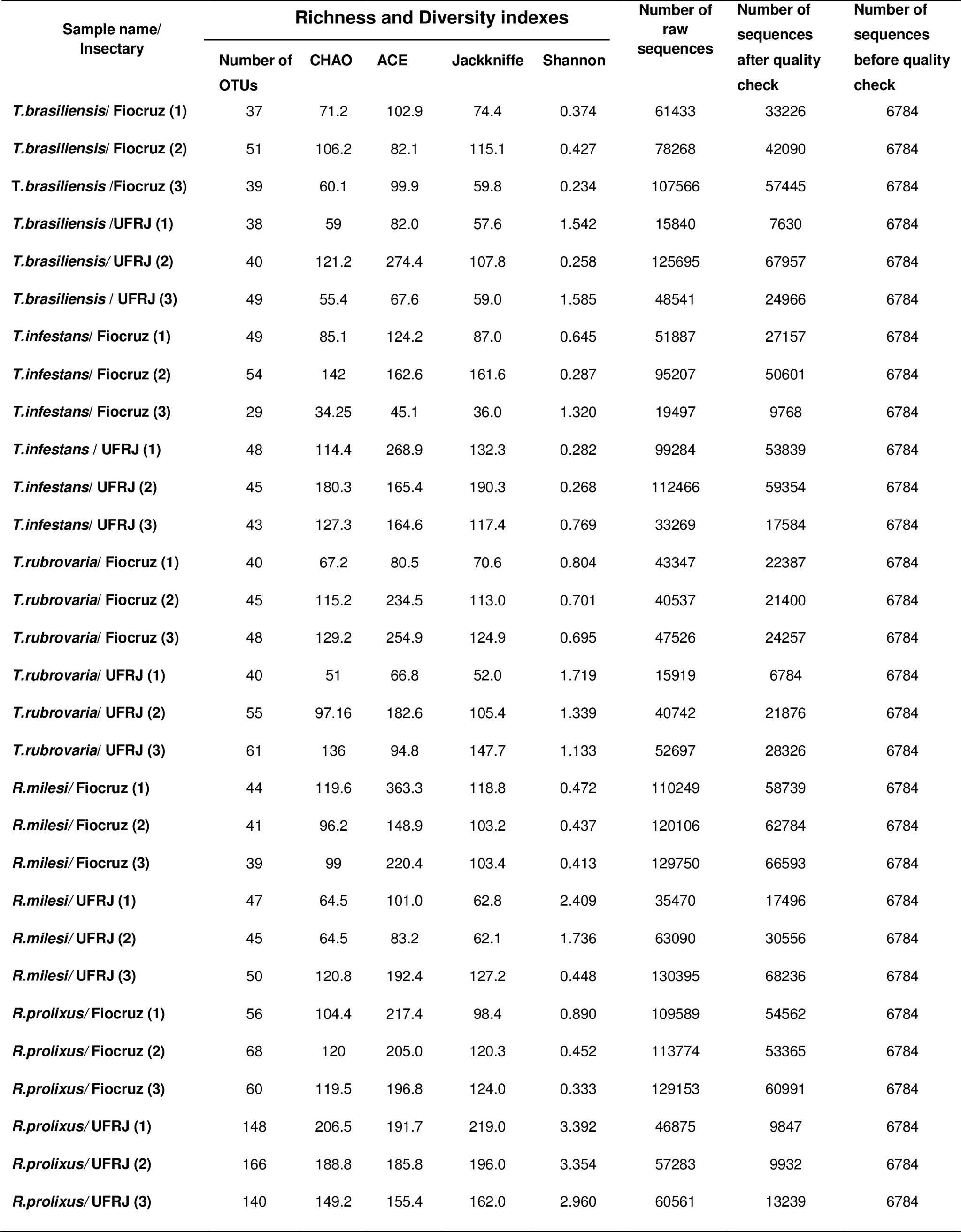

Supplement: S1 Table — (TIF) [file pntd.0006739.s005.tif]
